# Peroxiredoxin Ⅲ mitigates mitochondrial H_2_O_2_-mediated damage and supports quality control in cardiomyocytes under hypoxia-reoxygenation stress

**DOI:** 10.1016/j.redox.2025.103799

**Published:** 2025-08-05

**Authors:** Ji Won Park, Seong Keun Sonn, Byung-Hoon Lee, Goo Taeg Oh, Tong-Shin Chang

**Affiliations:** a College of Pharmacy, Seoul National University, 1 Gwanak-ro, Gwanak-gu, Seoul, 08826, Republic of Korea; b Heart-Immune-Brain Network Research Center, Department of Life Science, Ewha Womans University, Seoul, 03760, Republic of Korea; c Research Institute of Pharmaceutical Sciences, Seoul National University, 1 Gwanak-ro, Gwanak-gu, Seoul, 08826, Republic of Korea

**Keywords:** Peroxiredoxin Ⅲ, Mitochondrial oxidative stress, Hypoxia/reoxygenation injury, Mitophagy dysfunction, Cardiomyocyte injury

## Abstract

Peroxiredoxin Ⅲ (PrxⅢ) is a mitochondria-localized peroxidase that plays a key role in detoxifying hydrogen peroxide (H_2_O_2_) and preserving organelle homeostasis. While its antioxidant function is well established under physiological conditions, the role of PrxⅢ in the context of cardiac hypoxia/reoxygenation (H/R) injury remains incompletely understood. In this study, we investigated the protective function of PrxⅢ in cardiomyocytes exposed to H/R stress, a widely used *in vitro* model to mimic ischemia/reperfusion injury. Using H9c2 cells and primary neonatal rat cardiomyocytes, we found that PrxⅢ knockdown significantly increased mitochondrial H_2_O_2_ accumulation, leading to excessive mitochondrial fragmentation, impaired mitophagy, and reduced cell survival following H/R. Western blot analysis revealed that mitophagy regulators Parkin and BNIP3 were upregulated under moderate oxidative stress but were markedly suppressed in PrxⅢ-deficient cells after H/R, indicating that mitophagy activation is sensitive to the degree of oxidative stress. These findings were confirmed *in vivo* using mt-Keima transgenic mice, which showed significantly reduced mitophagic flux in PrxⅢ knockout hearts subjected to ischemia/reperfusion. In addition, PrxⅢ loss impaired lysosomal acidification and proteolytic activity, further contributing to defective autophagic flux. Re-expression of PrxⅢ restored mitochondrial morphology, mitophagy activity, and lysosome function, highlighting its central role in maintaining mitochondrial quality control (MQC). Collectively, our results demonstrate that PrxⅢ mitigates mitochondrial oxidative damage and preserves MQC by coordinating mitochondrial dynamics, mitophagy, and lysosomal integrity. These findings suggest that PrxⅢ may serve as a promising therapeutic target for preventing cardiac injury induced by oxidative stress during ischemia/reperfusion.

## Graphical abstract

**Peroxiredoxin III plays a protective function against hypoxia/reoxygenation-induced cardiotoxicity**.Moderately excess mitochondrial H_2_O_2_ accumulate when Prx III is depleted, disrupting mitochondrial dynamics and balance, triggering mitophagy to relieve oxidative stress. High accumulation of mitochondrial H_2_O_2_ in Prx III-knockdown cardiomyocytes under H/R stress induces mitochondrial fragmentation, disrupts mitochondrial dynamics, and blocks mitophagy, ultimately resulting in apoptosis.

**Figure:**
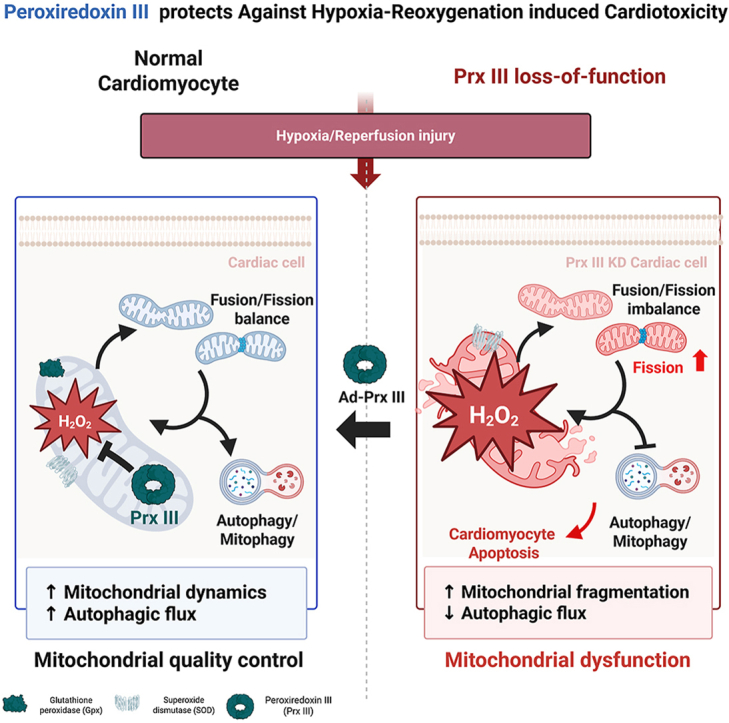
Graphical abstract created with BioRender.com under an Academic License. Created in BioRender. Park, J. (2025) https://BioRender.com/1ebmr6i.

## Abbreviation

8-OHdG: 8-hydroxy-2′-deoxyguanosine
10-NAO: 10-N-nonylacridine orange
Ad-GFP-LC3: recombinant adenoviral vector encoding GFP-tagged LC3
Ad-GFP-mCherry-LC3: recombinant adenoviral vector encoding tandem fluorescent-tagged LC3
Ad-mitoCatalase: recombinant adenoviral vector encoding human catalase fused with a mitochondrial leader sequence
Ad-PrxⅢ: recombinant adenoviral vector encoding the full-length human PrxⅢ cDNA
Atg: autophagy related gene
BNIP3: Bcl2 adenovirus e1B 19 kDa interacting protein 3
DAPI: 4′,6-diamidno-2-phenylindole
Drp1: dynamin-related protein 1
FUNDC1: FUN14 domain-containing 1
GAPDH: glyceraldehyde 3-phosphate dehydrogenase
Gpx: glutathione peroxidase
HIF-1α: hypoxia-inducible factor-1 α
H/R: hypoxia/reoxygenation
IHD: ischemic heart disease
I/R: ischemia/reperfusion
KO: knockout
LAMP1: lysosome-associated membrane protein 1
LC3: light-chain 3
Mfn: mitofusin
MI: myocardial infarction
MiNA: mitochondrial network analysis
MitoPY-1: mitochondria peroxy-yelow-1
MOI: multiplicities of infection
MQC: mitochondrial quality control
mtDNA: mitochondrial DNA
OPA1: optic atrophy 1
PARP: poly(ADP-ribose) polymerase
PI: propidium iodide
PINK1: PTEN-induced kinase 1
PO-1: peroxy orange-1
Prx: peroxiredoxin
RFI: relative fluorescence intensity
ROS: reactive oxygen species
RT: room temperature
siRNA: small interfering RNA
SOD: superoxide dismutase
TEM: transmission electron microscopy
Trx: thioredoxin
WT: wild-type
*ΔΨ*_m_: mitochondrial membrane potential

## 1 Introduction

Ischemic heart disease (IHD) is a leading cause of death, with an increasing prevalence worldwide. According to a 2020 epidemiological report, the global incidence of IHD is projected to rise to 1,845 cases per 100,000 individuals by 2030 [1]. IHD results from the blockage of coronary arteries, which compromises blood flow and causes oxygen and nutrient deprivation in myocardial tissue [2]. This ischemic damage can lead to myocardial infarction, angina pectoris, and heart failure [3]. Over the past five years, the clinical and economic burden of IHD has grown significantly, including a 49 % increase in treatment-related healthcare costs [4], highlighting the urgent need for improved therapeutic strategies.

Current treatment strategies focus on restoring coronary perfusion via pharmacological or surgical reperfusion therapies. However, the restoration of blood flow paradoxically exacerbates myocardial injury—a phenomenon known as ischemia/reperfusion (I/R) injury. This additional damage is attributed to oxidative stress and calcium overload during the reoxygenation phase, leading to mitochondrial dysfunction and cardiomyocyte death [[5], [6], [7]]. Reactive oxygen species (ROS) generated upon reactivation of the mitochondrial electron transport chain are considered the most critical mediators of I/R injury. Simultaneously, cytosolic calcium accumulation and pH normalization promote mitochondrial calcium influx and opening of the mitochondrial permeability transition pore, further amplifying ROS generation and cell death signaling [7,8]. Therefore, limiting oxidative damage by modulating mitochondrial ROS levels has emerged as a potential therapeutic strategy in I/R injury. *In vitro*, hypoxia/reoxygenation (H/R) is widely used to recapitulate the molecular and cellular features of I/R injury, providing a reliable model for mechanistic studies of oxidative stress and mitochondrial dysfunction.

The heart requires considerable energy to sustain contraction and relaxation, and mitochondria occupy approximately 25–30 % of cardiomyocyte volume [9,10]. Therefore, mitochondrial damage resulting from I/R injury is a primary mechanism of cardiac dysfunction. Mitochondria produce ATP via oxidative phosphorylation, but this process also generates ROS, as approximately 1 % of electrons leak from the electron transport chain during respiration [11,12]. As a result, mitochondria are not only the main source of cellular energy but also the major site of intracellular ROS generation [13]. ROS include superoxide anion (O_2_•^-^), hydrogen peroxide (H_2_O_2_), and hydroxyl radical (•OH), and while physiological levels of ROS serve signaling roles, excessive ROS induce oxidative stress and cellular injury [14].

Among ROS, H_2_O_2_ is of particular importance due to its chemical stability and uncharged nature, which allow it to diffuse across membranes and propagate oxidative stress. While H_2_O_2_ can act as a signaling molecule under physiological conditions, but excessive accumulation leads to cellular damage either directly or through conversion into more reactive species such as hydroxyl radicals via the Fenton reaction [15,16]. In mitochondria, superoxide anions generated by the electron transport chain are rapidly converted to H_2_O_2_ by superoxide dismutases (SODs), primarily SOD2 in the matrix and SOD1 in the intermembrane space [17]. Although this enzymatic dismutation mitigates superoxide toxicity, it shifts the oxidative burden toward H_2_O_2_, making its efficient removal essential for maintaining redox balance.

Several enzymes, including glutathione peroxidases (Gpx1 and GPx4) and peroxiredoxins (PrxⅢ and PrxV) are involved in mitochondrial H_2_O_2_ detoxification [18]. However, evidence suggests that PrxⅢ plays a dominant role in this process. PrxⅢ, a typical 2-Cys peroxiredoxin localized exclusively in the mitochondrial matrix, exhibits high expression levels and superior catalytic efficiency for H_2_O_2_ due to its low *K*_m_ and high turnover rate. These properties enable PrxⅢ to serve as the dominant mitochondrial H_2_O_2_ scavenger, responsible for approximately 90 % of its elimination in many cell types [19,20].Therefore, the functional integrity of PrxⅢ is critical for limiting mitochondrial oxidative stress and preventing H_2_O_2_-mediated damage under pathological conditions.

Mitochondrial quality control (MQC) is also essential for maintaining mitochondrial function, particularly under stress conditions [21]. The MQC system includes mitochondrial fusion, fission, and mitophagy, which collectively regulate mitochondrial morphology and function [22]. Fusion promotes the mixing of mitochondrial contents, fission segregates damaged regions, and mitophagy eliminates dysfunctional mitochondria through selective autophagy [23,24]. Under physiological conditions, these processes operate in a tightly regulated balance. However, severe or prolonged oxidative stress can disrupt MQC, leading to mitochondrial dysfunction and cell death. Although recent studies suggest that MQC represents a promising therapeutic target in I/R injury, its precise role in ROS-mediated cardiomyocyte damage remains under debate [25,26].

The cytoprotective functions of PrxⅢ have been well documented in various cell types. Nonn et al. demonstrated that PrxⅢ prevents hypoxia- and H_2_O_2_-induced apoptosis in cancer cells [27]. Our previous studies also confirmed that PrxⅢ plays a critical role in removing mitochondrial H_2_O_2_ and suppressing ROS-induced apoptosis. In HeLa cells, PrxⅢ knockdown resulted in ROS accumulation and mitochondrial damage–induced apoptosis [19], while in HaCaT keratinocytes, PrxⅢ suppression led to elevated mitochondrial H_2_O_2_ levels following UVB exposure, resulting in increased mitochondria-mediated apoptosis [28]. However, the role of PrxⅢ in regulating mitochondrial ROS and maintaining mitochondrial quality in cardiomyocytes under H/R stress remains unclear.

In this study, we investigated whether PrxⅢ protects cardiomyocytes from H/R-induced injury by limiting mitochondrial H_2_O_2_ accumulation and preserving mitochondrial function. In addition to analyzing oxidative damage, we assessed alterations in mitochondrial dynamics, mitophagic flux, and cardiomyocyte viability. Our results suggest that PrxⅢ plays a protective role in cardiomyocytes under H/R stress by regulating mitochondrial ROS levels and supporting MQC pathways.

## 2 Materials & methods

### 2.1 Reagents and antibodies

FBS and antibiotic-antimycin were from Gibco (NY, USA); poly-d-lysine and puromycin (P8920, P8833) (Sigma-Aldrich, St. Louis, MO, USA); Lipofectamine 2000, JC-1 (T3168), Mitotracker Red CMX (M7512), Lysosensor Green DND-189 (L7535) and DQ-BSA (D12051) were purchased from Invitrogen (CA, USA); 10-N-nonylacridine orange (10-NAO, A1372) were purchased from Molecular Probes (Eugene, OR, USA); mitochondria peroxy-yelow-1 (MitoPY-1, 4428) and peroxy orange-1 (PO-1, 4944) were from Tocris Biosciences (Bristol, UK); Magic Red kit (938) was from Immuno Chemistry Technology (ICT, Davis, CA, USA); 4’,6-diamidno-2-phenylindole (DAPI) (62248) was from Thermo Fisher Scientific (Waltham, MA, USA).

The primary antibodies against poly(ADP-ribose) polymerase (PARP) (9542), cleaved caspase-3 (9661s), caspase-9 (9508), pDrp1 (Ser616; 3455s), pDrp1 (Ser637; 6319s), light-chain 3B (LC3B) (2775s), SQSTM1/p62 (5114T), lysosome-associated membrane protein 1 (LAMP1) (9091s), hypoxia-inducible factor-1 α (HIF-1α) (14179s), thioredoxin 2 (Trx2) (14907s) and HSP60 (12156) were purchased from Cell Signaling Technology (CST, Danvers, MA, USA); 8-hydroxy-2′-deoxyguanosine (8-OHdG) (SC66036), Parkin (PRK8l SC32282), Bcl2 adenovirus e1B 19 kDa interacting protein 3 (BNIP3) (SC56167), Drp1 (SC271583), and mitofusin 1 (Mfn1) (SC166644) were purchased from Santa Cruz Biotechnologies (Santa Cruz, Dallas, TX, USA); optic atrophy 1 (OPA1) (ab42364) was purchased from Abcam (Cambridge, UK); cytochrome C (556433) was purchased from BD Pharmingen (San Jose, CA, USA); catalase (LF-PA0060), Gpx4 (LF-PA0055), SOD2 (LF-PA0021), β-actin (LF-PA0207) and PrxⅢ (mono; LF-MA0043) were purchased from Ab Frontier (Seoul, Republic of Korea); glyceraldehyde 3-phosphate dehydrogenase (GAPDH) (MAB374) was purchased from Chemicon (Jincheon, Republic of Korea); The secondary antibodies against anti-mouse IgG HRP (474–1806) and anti-rabbit IgG HRP (5220-0458) were purchased from Seracare (Milford, MA, USA).

### 2.2 Cell culture and transfection

H9c2 rat cardiomyoblast cell (CRL-1446; ATCC, Manassas, VA, USA), was maintained in DMEM (SH30022.01; Hyclone, Logan, UT, USA) supplemented with 10 % FBS and 1 % antibiotic-antimycin in a humidified 5 % CO_2_ atmosphere.

### 2.3 Establishment of H9c2 cells stably expressing short hairpin RNA targeting PrxⅢ

To generate a plasmid expressing a short hairpin RNA specific for rat PrxⅢ, two complementary oligonucleotides targeting the 5′-AAGAGCUGAGUCUCGACGACU-3′ sequence within the open reading frame of rat PrxⅢ mRNA (Genotech, Daejeon, Republic of Korea) were synthesized;

5′-GATCCCCGAGCTGAGTCTCGACGACTTTCAAGAGAAGTCGTCGAGACTCAGCTCTTTTTA-3′

5′-AGCTTAAAAAGAGCTGAGTCTCGACGACTTCTCTTGAAAGTCGTCGAGACTCAGCTCGGG-3’ The oligonucleotides were annealed and ligated into the pSUPER-puro vector (VEC-pBS-0008; OligoEngine, Seattle, WA, USA), resulting in the short hairpin RNA-expressing construct pSUPER-siPrxⅢ.

H9c2 cells were transfected with either pSUPER-siPrxⅢ or control pSUPER vectors using Lipofectamine 2000 Transfection Reagent (11668019; Invitrogen, Carlsbad, CA, USA) according to the manufacturer's protocol. After 48 h, cells were selected with puromycin (1 μg/mL), and puromycin-resistant clones were isolated and expanded. Stable knockdown of PrxⅢ protein expression was confirmed by immunoblot analysis.

### 2.4 Adenoviral-mediated expression

To overexpress mitochondria-targeted human catalase, we utilized a recombinant adenoviral vector encoding human catalase fused with a mitochondrial leader sequence (Ad-mitoCatalase), which was kindly provided by Dr. Arthur I. Cederbaum, as described previously [19]. To overexpress human PrxⅢ, we used a recombinant adenoviral vector encoding the full-length human PrxⅢ cDNA (Ad-PrxⅢ), which was constructed and packaged by VectorBuilder (vector ID: VB220222-1017kpd, Chicago, IL, USA). As a negative control, we employed an adenoviral vector carrying a non-coding stuffer sequence (Ad-Stuffer, vector ID: VB220301-1013hty, VectorBuilder).

To monitor autophagosome formation and autophagic flux, cells were transduced with recombinant adenoviral vectors encoding GFP-tagged LC3 (Ad-GFP-LC3) or tandem fluorescent-tagged LC3 (Ad-GFP-mCherry-LC3), which were kindly provided by X.M. Yin and J.S. Kim, respectively as described previously [29].

All adenoviral transductions were performed in serum-free medium for 4 h, after which the medium was replaced with complete growth medium and cells were further incubated for 20 h before downstream analysis.

### 2.5 H/R stress

Hypoxia was induced by incubating H9c2 cells in a hypoxia chamber (MIC-101; IBscience, Daejeon, Republic of Korea). The cells were maintained at 37 °C with a humidified atmosphere of 5 % CO_2_, 2 % O_2_, and 93 % N_2_ in d-glucose and serum-free DMEM. Reoxygenation was subjected to maintain cells with DMEM in 37 °C with a humidified atmosphere of 5 % CO_2_ followed by hypoxic stress.

### 2.6 Flow cytometry

To analyze cell death, cells were resuspended in annexin binding buffer and labeled with annexin V-FITC and propidium iodide (PI) at 25 °C for 15 min according to the manufacturer's instructions (FITC Annexin V Apoptosis Detection Kit I, (556547; BD Biosciences, San Jose, CA, USA). To evaluate mitochondrial membrane potential (*ΔΨ*_m_) changes, cells were incubated with JC-1 (2 μM) at 37 °C for 20 min, and the shifts in red fluorescence ratio of red/green were measured. To measure cytoplasmic or mitochondrial H_2_O_2_ levels, cells were incubated with PO-1 (5 μM) or MitoPY-1 (5 μM) at 37 °C for 15 min. To detect mitochondrial lipid oxidation [30], cells were incubated with 10-NAO (5 μM) at 37 °C for 15 min.

A FACSCalibur flow cytometer (BD Biosciences, San Jose, CA, USA) was used for all analyses, with a minimum of 2 × 10^5^ cells per sample for each measurement. Cell populations were identified and gated based on forward scatter and side scatter characteristics. The relative change in fluorescence was analyzed with FlowJo software 10.9 (BD Biosciences).

### 2.7 Subcellular fractionation

Subcellular fractionation was performed as previously described [31] with slight modifications. Cells were homogenized in hypotonic buffer containing protease inhibitors, and isotonicity was restored by addition of hypertonic sucrose buffer. After removal of nuclei and unbroken cells by low-speed centrifugation, the post-nuclear supernatant was centrifuged at 15,000×*g* to obtain the mitochondria-enriched heavy membrane fraction. The resulting supernatant was collected as the cytosolic fraction. The purity of mitochondrial and cytosolic fractions was confirmed by immunoblotting using HSP60 and β-actin as markers, respectively.

### 2.8 Mitochondrial protein oxidation analysis

For the detection of cytosol and mitochondrial protein oxidation, protein carbonylation was examined after subcellular fractionation. The carbonyl protein content in cytosol fraction and mitochondria-enriched heavy membrane fraction was determined using a Protein Carbonyl Colorimetric Assay Kit (#10005020, Cayman Chemical Company, Ann Arbor, MI, USA) following the manufacturer's instructions. The result was presented as nmol protein carbonyl content per mg total protein.

### 2.9 Mitochondrial cytochrome *c* release

After subcellular fractionation, the cytosolic fraction was subjected to immunoblot analysis for cytochrome *c*.

### 2.10 Immunoblot analysis

Cell lysates were prepared with lysis buffer containing 20 mM HEPES (pH 7.0), 150 mM NaCl, 1 % TritonX-100, 10 % glycerol, 2 mM EGTA, 1 mM EDTA, 20 mM glycerol 2-phosphate, 1 mM Na_3_VO_4_, 1 μg/ml aprotinin, 1 μg/ml leupeptin and 1 mM AEBSF and centrifuged at 12,500×*g* for 10 min at 4 °C The protein concentration of each cell lysate was measured by Bradford assay. The constant amounts of cell lysates were loaded on appropriate SDS-polyacrylamide gel for electrophoresis and transferred to nitrocellulose membrane (Whatman, Maidstone, UK). Then, membranes were blocked with 5 % BSA for 1 h at RT. After blocking, blots were probed with primary antibodies for overnight at 4 °C, followed by secondary antibodies in 5 % skim milk for 1 h at RT. After probing, the blots were subjected to ECL solution (WESTSAVE-up; Ab frontier) and detected by Amersham Imager 680 (GE Healthcare). The density of bands was quantified with ImageJ/Fiji software (NIH, Bethesda, MD, USA).

### 2.11 Immunocytochemistry

For the detection of mitochondrial DNA (mtDNA) oxidation, immunostaining of 8-OHdG in mitochondria was used [32]. To detect the amount of 8-OHdG in mitochondria, MitoTracker Red CMXROS (30 nM) was added to the seeded H9c2 cells at 37 °C for 20 min. The cells were then washed with PBS and fixed with 4 % paraformaldehyde at 4 °C for 15 min. Then, the cells were permeabilized using 0.1 % Triton X-100 at RT for 15 min and washed with PBS before being blocked with 3 % (w/v) BSA in PBS for 1 h at RT. Subsequently, cells were incubated with primary antibodies against 8-OHdG at a 1:300 dilution overnight at 4 °C. After three washes in the blocking solution, the cells were incubated with the secondary antibodies, Alexa Fluor 488 goat anti-mouse IgG (H + L) (A-11001, Thermo Fisher Scientific, Waltham, MA, USA) for 2 h at RT and then washed three more times in blocking solution. Finally, the cells were stained with DAPI before being imaged. Fixed cells were examined with the TCS8 confocal microscopy (Leica, Wetzlar, Germany). Co-localization appeared as a yellow color due to the overlay of green and red signals. Images were analyzed using ImageJ/Fiji software.

### 2.12 Mitochondrial fragmentation measurement

Cells were seeded in 6-well plates with poly-d-lysine-coated cover glass. After H/R stress, cells were stained with MitoTracker Red (30 nM) for 15 min in 37 °C shaded from light, washed with PBS, and fixed with 4 % paraformaldehyde solution for 15 min. Then, Images were taken with TCS8 confocal microscopy and analyzed with ImageJ/Fiji software with the mitochondrial network analysis (MiNA) program. Mitochondrial networks were analyzed using the mean branch length, median branch length and the aspect ratios supplied by the MiNA program [33]. Mitochondrial fission and fusion proteins were determined by immunoblot analysis regarding Drp1, phopho-Drp1 (Ser616), phospho-Drp1 (Ser637), Mfn1, and OPA 1.

### 2.13 Autophagic flux analysis *in vitro*

Autophagic flux analysis was performed as described previously [34]. Autophagosome proteins were measured by immunoblot analysis regarding LC3B and SQSTM1/p62. Cells plated in 6-well plates were grown for 24 h on poly-d-lysine-coated cover glass in DMEM without antibiotic-antimycotic. Cells were infected with Ad-GFP-LC3 or Ad-GFP-mCherry-LC3 adenovirus. After treatment, the cover glass was washed with PBS and fixed with 4 % paraformaldehyde solution for 15 min. The images were taken with the TCS8 confocal microscopy, and puncta were measured using ImageJ/Fiji software.

### 2.14 Lysosomal damage analysis

Immunofluorescence analysis was performed as described previously [29]. For visualization of the lysosome, fixed samples were exposed to a blocking solution and incubated for 30 min at RT with antibodies to LAMP1. The cells were washed three times with PBS and then incubated for 30 min at RT with Alexa Fluor 546-conjugated secondary antibodies. For DQ-BSA assay, cells were preloaded with 10 μg/ml DQ-BSA Red in prewarmed media for 12 h. They were washed with PBS and fixed with 4 % paraformaldehyde. For measurement of lysosomal acidity or activity, cells were loaded with Lysosensor Green DND-189 or Magic Red reagent (ICT; Immuno Chemistry Technologies) in a prewarmed media for 1 h after treatment. Then the cells were washed three times with PBS and fixed with 4 % paraformaldehyde. Images were captured with Leica TCS8 confocal microscope, and fluorescence intensity was quantified using ImageJ/Fiji software.

### 2.15 Primary cardiomyocyte isolation and culture

Primary cardiomyocytes were isolated from wild-type (WT) and PrxⅢ knockout (KO) mice on postnatal day 3 using the Pierce™ Primary Cardiomyocyte Isolation Kit (88281, Thermo Fisher Scientific, Waltham, MA, USA), according to the manufacturer's instructions as described previously [35]. Briefly, mouse pups were euthanized and hearts were rapidly harvested placed into ice-cold Hank's Balanced Salt Solution. After removal of connective tissues, hearts were minced and enzymatically digested using the provided enzyme solutions. Cardiomyocytes were gently dissociated and filtered through a 70 μm cell strainer to remove tissue debris. Isolated cardiomyocytes were collected by centrifugation and plated onto gelatin-coated culture dishes. Cells were cultured in complete Cardiomyocyte Medium (88287, Thermo Fisher Scientific, Waltham, MA, USA) supplemented with 10 % FBS and 1 % antibiotic-antimycin, and maintained at 37 °C in a humidified atmosphere with 5 % CO_2_. Cardiomyocytes were cultured for 7 days prior to analysis to confirm proper cell morphology and stabilization. After stabilization, cardiomyocytes were subjected to H/R conditions as described above.

### 2.16 Mouse myocardial infarction model and electron microscopy

All animal care procedures and experiments were performed in compliance with protocols approved by the Institutional Animal Care and Use Committee of Ewha Womans University. WT and PrxⅢ KO mice (10–12 weeks old) underwent cardiac surgery under anesthesia with isoflurane. The left anterior descending coronary artery was permanently occluded using 7-0 silk suture to induce myocardial infarction (MI).

For transmission electron microscopy (TEM) analysis, heart tissue was harvested from 10-week–old WT and *Prx*Ⅲ KO mice. The collected tissues were immediately fixed in a solution containing 2 % glutaraldehyde-paraformaldehyde in 0.1 M PBS (pH 7.4) for 2 h at RT, followed by washing three times (30 min each) in 0.1 M PBS (pH 7.4). Subsequently, tissues underwent secondary fixation with 1 % osmium tetroxide (OsO_4_) in 0.1 M PBS (pH 7.4) for 2 h and were dehydrated stepwise in graded ethanol solutions (50 %, 60 %, 70 %, 80 %, 90 %, 95 %, and 100 %). After dehydration, tissues were incubated with propylene oxide and then embedded in fresh Poly/Bed 812 resin (Polysciences, Warrington, PA, USA). Resin polymerization was conducted at 60 °C for 24 h.

Semi-thin (300 nm) sections were initially obtained using an Ultracut UCT Ultramicrotome (Leica Microsystems, Vienna, Austria), stained with toluidine blue, and examined under a light microscope (Olympus BX40, Tokyo, Japan) to identify regions of interest. Subsequently, ultra-thin sections (80 nm) were prepared and contrasted by double staining with 7 % uranyl acetate and lead citrate for 20 min. Electron microscopy observation was carried out using a JEM-1011 transmission electron microscope (JEOL, Tokyo, Japan) operated at an acceleration voltage of 80 kV.

### 2.17 Autophagic flux analysis *in vivo* using mitochondria-targeted Keima mice

To measure mitophagy flux *in vivo*, we utilized cardiac-specific mitochondria-targeted Keima (mt-Keima) transgenic mice, as previously established [35]. mt-Keima is a pH sensitive fluorescent protein that exhibits different excitation spectra depending on pH: excitation at 440 nm detects mitochondria in neutral environments (cytosol or mitochondria), whereas excitation at 561 nm specifically identifies mitochondria in acidic lysosomes after mitophagic flux [36,37].

Briefly, adult WT/mt-Keima and PrxⅢ KO/mt-Keima mice (10 weeks old) underwent MI (30 min) followed by reperfusion (24 h). Heart tissues were rapidly isolated, snap-frozen, sectioned, and imaged using confocal microscopy equipped with appropriate excitation filters for 440 nm (neutral pH) and 561 nm (acidic pH). The degree of mitophagy was quantified by analyzing the ratio of fluorescent intensity at 561 nm excitation (acidic lysosomal mitochondria) to 440 nm excitation (neutral mitochondria) using ImageJ/Fiji software. A reduced 561/440 nm fluorescence ratio indicated impaired mitophagic flux.

### 2.18 Statistics

Statistical analysis and sample size calculations were performed using GraphPad PRISM (version 10.1.2, GraphPad Software, San Diego, CA, USA). The effect size was estimated based on preliminary data or similar studies, with an effect size f = 0.4, a power of 0.8 and a significance level of 0.05. All experiments were independently replicated at least three to five times, *n* represents the number of biologically independent replicates for each experiment. All data are presented as the mean ± standard deviation (S.D.). Statistical significance was considered at a threshold of p-value <0.05.

Statistical significance between groups was primarily analyzed using two-way ANOVA, followed by Bonferroni's post hoc test. For experiments requiring comparisons between two groups only, statistical analysis was performed using Unpaired *t*-test.

## 3 Results

### 3.1 Validation of PrxⅢ knockdown model and characterization of H_2_O_2_ accumulation under H/R stress

**Fig. 1:**
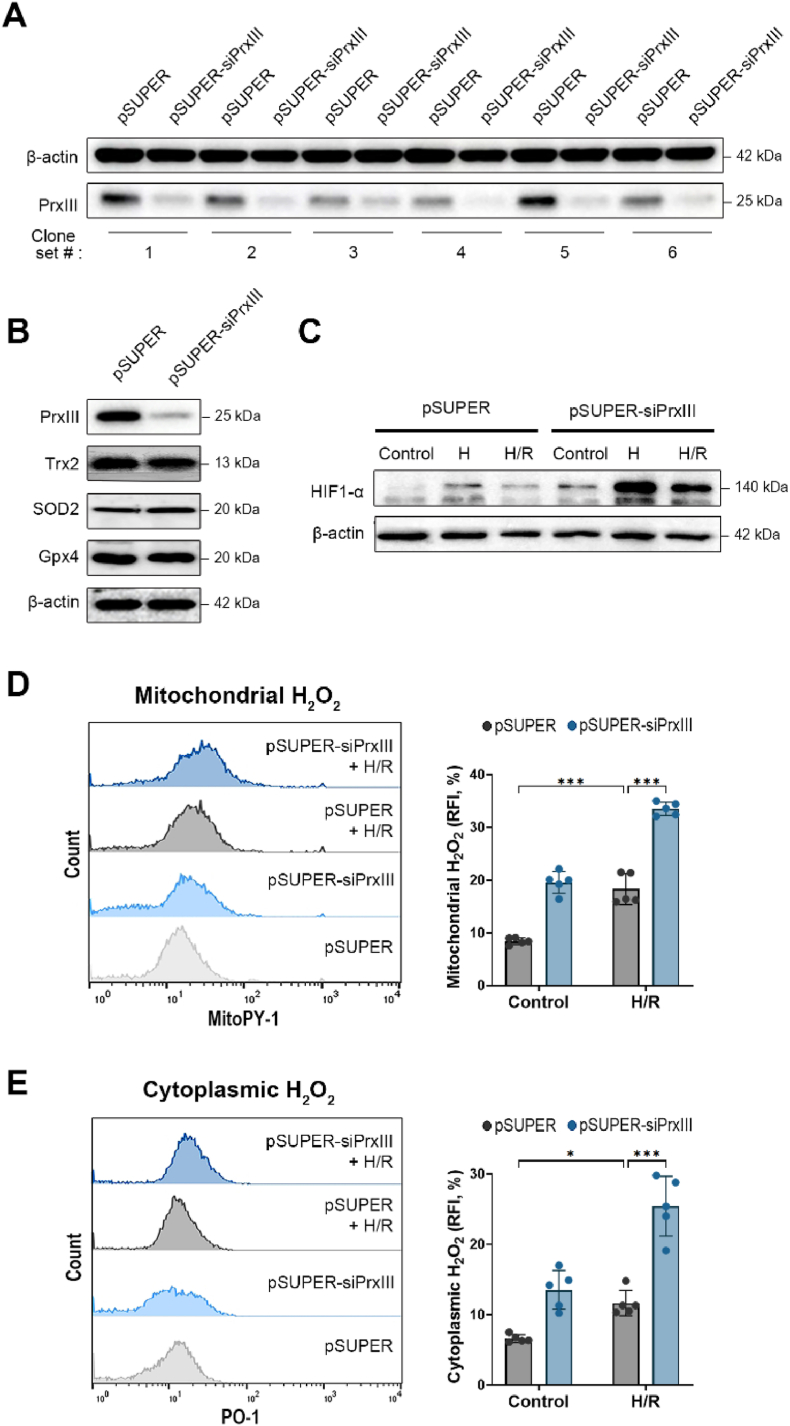
PrxⅢ depletion exacerbates mitochondrial and cytoplasmic H_2_O_2_ accumulation under hypoxia/reoxygenation (H/R) stress in cardiomyocytes. (A) Clone validation for PrxⅢ knockdown. H9c2 cardiomyocytes were transfected with either pSUPER (control) or pSUPER-siPrxⅢ vectors, and stable clones were selected by puromycin resistance. Western blotting was performed on six independent clone sets to evaluate PrxⅢ expression, with β-actin used as a loading control. (B) Protein expression of mitochondrial antioxidant enzymes (Trx2, SOD2, and Gpx4) in pSUPER and pSUPER-siPrxⅢ cells under basal conditions. (C) Validation of hypoxia/reoxygenation conditions using HIF-1α protein levels. Cells were subjected to normoxia (Control), hypoxia for 1 h (H), or hypoxia followed by 4 h of reoxygenation (H/R), and lysates were immunoblotted for HIF-1α and β-actin. (D) Mitochondrial H_2_O_2_ was measured using MitoPY-1 and quantified by flow cytometry. (E) Cytoplasmic H_2_O_2_ was detected using the PO-1 fluorescent probe and analyzed by flow cytometry. Representative histograms (left) and quantification of relative fluorescence intensity (RFI, %) (right) are shown for D and E. All data are expressed as mean ± S.D. from independent biological replicates (n = 5). Statistical significance was determined using two-way ANOVA followed by Bonferroni's post hoc test. ∗p < 0.05 and ∗∗∗p < 0.001.

To investigate whether PrxⅢ protects cardiomyocytes from oxidative stress during H/R injury by limiting mitochondrial H_2_O_2_ accumulation, we established a stable PrxⅢ-knockdown H9c2 cell model using a short hairpin RNA approach. H9c2 cardiomyocytes stably transfected with either pSUPER or pSUPER-siPrxⅢ vectors were designated as pSUPER and pSUPER-siPrxⅢ cells, respectively. Six puromycin-resistant clones were screened by Western blotting, and Clone set 5 showing a marked reduction of PrxⅢ protein levels in pSUPER-siPrxⅢ cells compared to pSUPER controls was selected for further experiments (Fig. 1A). To verify the specificity of the knockdown system, we confirmed that the expression of other major mitochondrial antioxidant enzymes—including Trx2, SOD2, and Gpx4—remained unchanged in pSUPER-siPrxⅢ cells compared to controls (Fig. 1B), suggesting that PrxⅢ depletion does not trigger compensatory upregulation of these antioxidant enzymes.

Based on time-course analyses of mitochondrial H_2_O_2_ levels (Supplementary Fig. S1A), we selected 1 h of hypoxia followed by 4 h of reoxygenation as the standard H/R condition. We next validated the cellular H/R model by monitoring HIF-1α protein levels, a canonical marker of hypoxic stress. HIF-1α expression was significantly elevated after 1 h of hypoxia and declined following 4 h of reoxygenation in both pSUPER and pSUPER-siPrxⅢ cells (Fig. 1C), consistent with the well-established mechanism of oxygen-dependent degradation via prolyl hydroxylation [38,39]. These data confirm the validity of our *in vitro* H/R protocol and the successful establishment of a PrxⅢ knockdown cell model.

To assess the redox phenotype of PrxⅢ-deficient cardiomyocytes, we quantified mitochondrial and cytosolic H_2_O_2_ levels following H/R. MitoPY-1, a mitochondria-targeted fluorescent probe that specifically detects H_2_O_2_ through boronate-to-phenol conversion [40], revealed that mitochondrial H_2_O_2_ levels were significantly elevated after H/R in both groups, with a more pronounced increase in pSUPER-siPrxⅢ cells (Fig. 1D). Since uncharged H_2_O_2_ readily diffuses across mitochondrial membranes into the cytosol, we also evaluated intracellular H_2_O_2_ levels using the fluorescent probe PO-1. Consistent with mitochondrial findings, cytosolic H_2_O_2_ was also elevated following H/R and further increased in pSUPER-siPrxⅢ cells (Fig. 1E). These results demonstrate that PrxⅢ plays a central role in suppressing both mitochondrial and cytoplasmic H_2_O_2_ accumulation during H/R stress.

### 3.2 PrxⅢ depletion enhances mitochondrial macromolecular oxidative damage under H/R stress in cardiomyocytes

**Fig. 2:**
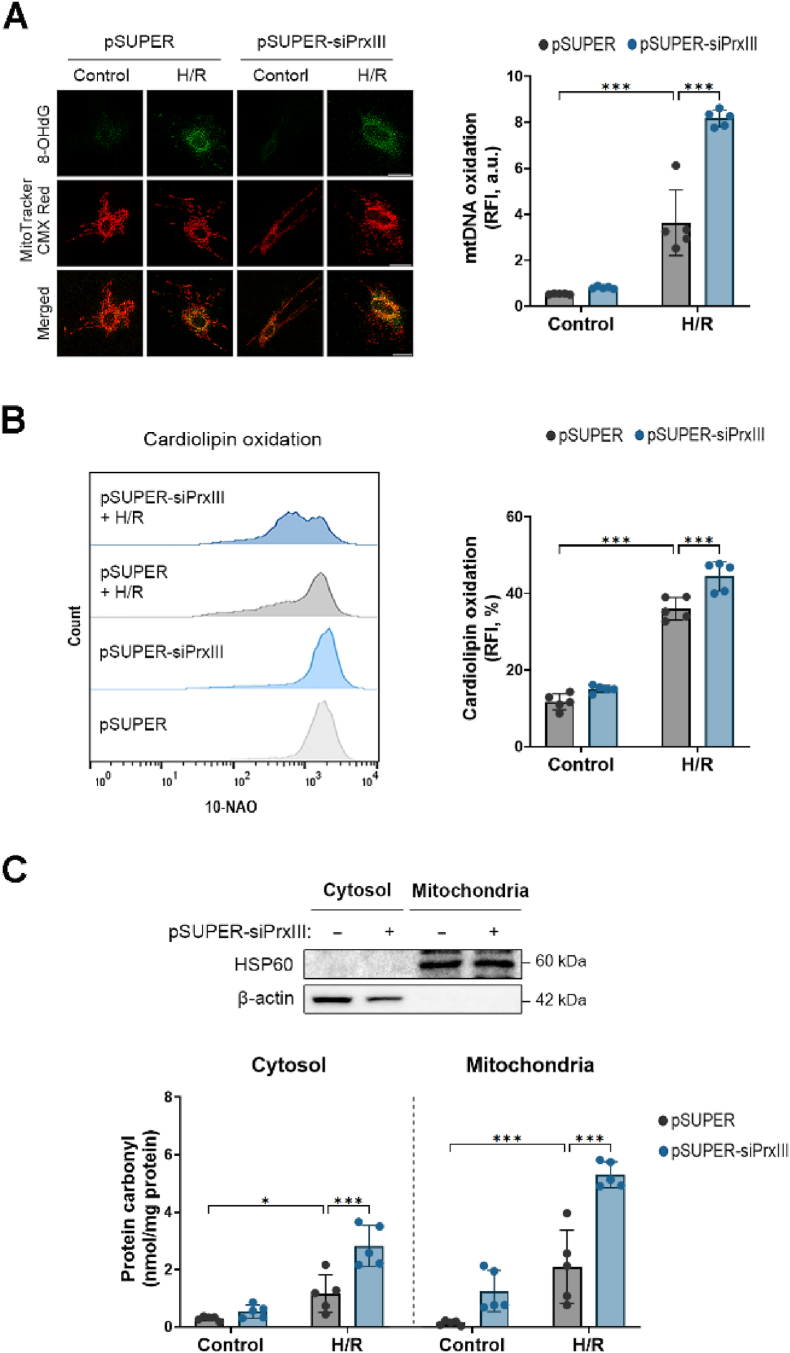
PrxⅢ depletion enhances mitochondrial macromolecule oxidative damage under H/R stress in cardiomyocytes. pSUPER and pSUPER-siPrxⅢ cells underwent 1 h of hypoxia and 4 h of reoxygenation (H/R). (A) Mitochondrial DNA (mtDNA) oxidation was assessed by immunofluorescence using anti-8-OHdG (green) antibody and MitoTracker CMX Red (red). Co-localization (yellow) indicates oxidized mtDNA. Five randomly selected microscopic fields per sample were analyzed and combined to represent one biological replicate (n = 5). Scale bar, 10 μm. (B) Mitochondrial lipid peroxidation was evaluated by flow cytometric analysis using 10-N-nonylacridine orange (10-NAO) as a cardiolipin oxidation probe. Representative histograms and quantification are shown. (C) Protein carbonylation was measured in cytosolic and mitochondrial fractions following subcellular fractionation. Purity of fractions was confirmed by immunoblotting with HSP60 (mitochondria) and β-actin (cytosol). Protein carbonyl content was quantified spectrophotometrically and normalized to protein concentration (nmol/mg protein). All data are presented as mean ± S.D. (n = 5). Statistical significance was determined using two-way ANOVA followed by Bonferroni's post hoc test. ∗p < 0.05 and ∗∗∗p < 0.001.

To assess the oxidative damage induced by mitochondrial H_2_O_2_, we measured the oxidation levels of key mitochondrial macromolecules, including DNA, lipids, and proteins. Oxidative damage to mtDNA was detected by co-staining with 8-OHdG and MitoTracker Red. Co-localization (yellow) was used as a readout for oxidized mtDNA. Confocal microscopy revealed increased mtDNA oxidation following H/R stress, and this signal was further elevated in PrxⅢ-depleted cells compared to controls (Fig. 2A).

Mitochondrial lipid peroxidation was assessed by flow cytometry using 10-NAO, a cardiolipin oxidation probe. Cardiolipin, a mitochondria-specific phospholipid located in the inner membrane, exhibited higher levels of oxidation after H/R, particularly in PrxⅢ-deficient cardiomyocytes (Fig. 2B).

Protein oxidation was evaluated in both cytosolic and mitochondrial fractions using a spectrophotometric carbonyl assay following subcellular fractionation. Fractionation quality was confirmed by immunoblotting with HSP60 (mitochondrial marker) and β-actin (cytosolic marker). Oxidative protein damage was increased in both compartments after H/R and was further exacerbated in PrxⅢ-depleted cells. Notably, the extent of protein oxidation was more pronounced in the mitochondrial fraction (Fig. 2C).

These data indicate that PrxⅢ depletion aggravates H/R-induced oxidative damage to mitochondrial DNA, lipids, and proteins in cardiomyocytes. Given this cumulative macromolecular damage, we next investigated whether mitochondrial integrity and quality control processes—such as fission, fusion, and autophagy—were also disrupted under H/R stress in the absence of PrxⅢ.

### 3.3 PrxⅢ depletion aggravates mitochondrial fragmentation following H/R stress in cardiomyocytes

**Fig. 3:**
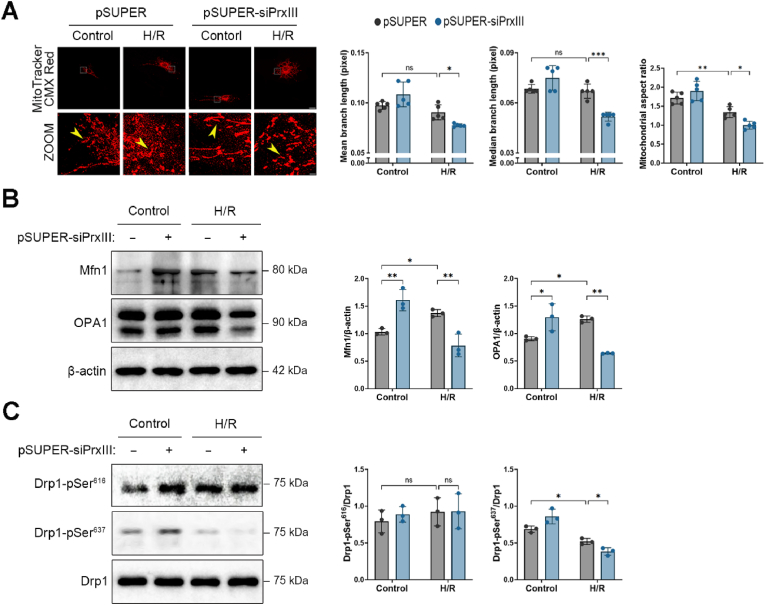
PrxⅢ depletion enhances mitochondrial fragmentation under H/R stress in cardiomyocytes. (A) Mitochondrial morphology was assessed in pSUPER and pSUPER-siPrxⅢ cells following 1 h of hypoxia and 4 h of reoxygenation (H/R). Cells were stained with MitoTracker CMX Red, and images were acquired by confocal microscopy. Yellow arrows indicate individual mitochondria. Zoomed images represent magnifications of boxed regions in the upper panels. Mitochondrial branch length (mean and median) and mitochondrial aspect ratio were quantified using the ImageJ MiNA and Analyze Particles tools. Five randomly selected microscopic fields per sample were analyzed and combined to represent one biological replicate (n = 5). Scale bars, 15 μm (upper) and 1 μm (lower). (B) Western blot analysis of mitochondrial fusion-related proteins Mfn1 and OPA1 was performed in control and PrxⅢ-deficient cells after H/R. β-actin was used as a loading control. (C) Western blot analysis of mitochondrial fission-associated Drp1 phosphorylation at Ser616 and Ser637. Total Drp1 was used for normalization. (B, C) Densitometric quantification was performed from three independent biological replicates (n = 3). All data are presented as mean ± S.D. Statistical significance was determined using two-way ANOVA followed by Bonferroni's post hoc test. ∗p < 0.05, ∗∗p < 0.01, ∗∗∗p < 0.001, and ns, p > 0.05 (not significant).

To investigate whether PrxⅢ depletion alters mitochondrial integrity under oxidative stress, we examined mitochondrial morphology following H/R exposure using MitoTracker CMX Red staining and confocal microscopy. In pSUPER-siPrxⅢ cells, mitochondrial branch length was significantly shortened after H/R, indicating increased mitochondrial fragmentation, whereas no such change was observed in pSUPER controls (Fig. 3A). This is consistent with the established role of oxidative stress in promoting mitochondrial fission and impairing fusion [21,26].

To complement these findings, we analyzed mitochondrial aspect ratios as an additional morphological parameter. A shift toward lower aspect ratio values—reflecting more rounded and fragmented mitochondria—was observed in PrxⅢ-deficient cells under H/R (Fig. 3A, far right). This quantitative metric further supports enhanced mitochondrial fission in the absence of PrxⅢ.

Since mitochondrial shape is dynamically regulated by the balance of fission and fusion processes, we next examined the expression of key regulatory proteins. Western blot analysis revealed that expression of the outer membrane fusion protein Mfn1 and the inner membrane protein OPA1, was moderately increased in PrxⅢ-depleted cells under normoxic conditions, but significantly decreased following H/R exposure (Fig. 3B). This suggests a possible compensatory upregulation of fusion pathways in response to basal oxidative stress, which fails under more severe oxidative conditions [41].

We then evaluated phosphorylation of Drp1, the key mitochondrial fission mediator. Phosphorylation at Ser637, which inhibits Drp1 translocation to mitochondria, was significantly reduced in PrxⅢ-deficient cells after H/R. In contrast, phosphorylation at Ser616, which promotes Drp1 recruitment and oligomerization, was slightly increased following H/R but did not significantly differ between groups (Fig. 3C). These findings align with prior reports showing that redox-dependent signaling regulates Drp1 activity primarily via Ser637 dephosphorylation under stress conditions [42].

Together, these findings indicate that PrxⅢ deficiency enhances mitochondrial fragmentation following H/R injury by downregulating mitochondrial fusion proteins and facilitating Drp1-dependent fission via loss of inhibitory Ser637 phosphorylation. This imbalance in mitochondrial dynamics may impair MQC and contribute to increased vulnerability to oxidative stress in PrxⅢ-deficient cardiomyocytes.

### 3.4 PrxⅢ depletion impairs autophagic flux following H/R stress in cardiomyocytes

**Fig. 4:**
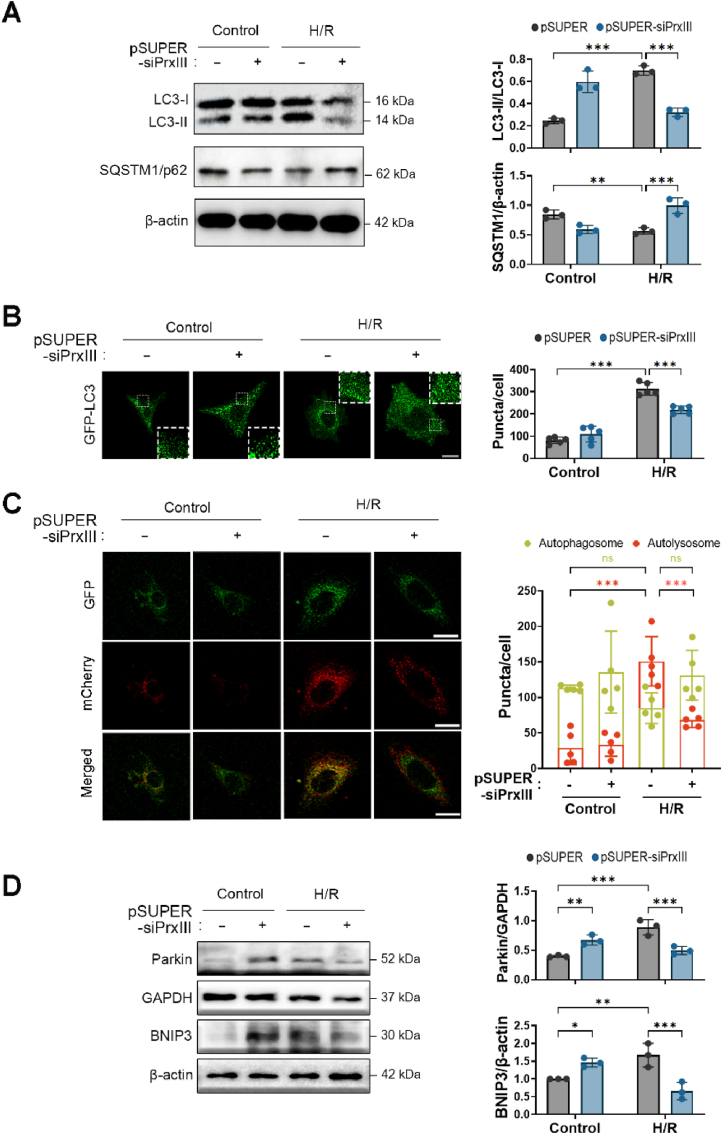
PrxⅢ depletion inhibits autophagosome formation and suppresses autophagy flux under H/R stress in cardiomyocytes. (A) pSUPER and pSUPER-siPrxⅢ cells were subjected to 1 h of hypoxia followed by 4 h of reoxygenation (H/R). Immunoblotting was performed to assess LC3 conversion (LC3-I to LC3-II) and SQSTM1/p62 expression. β-actin was used as a loading control. Densitometric quantification of protein expression was performed from three independent biological replicates (n = 3). (B) Cells were transduced with GFP-LC3 adenovirus and subjected to H/R. Representative confocal images show GFP-LC3 puncta. White boxes indicate zoomed regions. Scale bars, 10 μm (main) and 1 μm (insets). (C) Cells were transduced with mCherry-GFP-LC3 adenovirus and subjected to H/R. Representative images of autophagosomes (yellow puncta) and autolysosomes (red puncta) are shown. Scale bar, 10 μm. (B, C) Five randomly selected microscopic fields per sample were analyzed and combined to represent one biological replicate (n = 5). (D) Immunoblot analysis of Parkin and BNIP3 protein expression in H/R-treated cells. GAPDH and β-actin were used as loading controls, respectively. Densitometric quantification of protein expression was performed from three independent biological replicates (n = 3). All data are presented as mean ± S.D. Statistical significance was determined using two-way ANOVA followed by Bonferroni's post hoc test. ∗p < 0.05, ∗∗p < 0.01, and ∗∗∗p < 0.001.

To determine whether PrxⅢ deficiency affects autophagy under H/R conditions, we first evaluated the formation of autophagosomes using LC3B immunoblotting. Conversion of LC3-I to the lipidated LC3-II form, a marker of autophagosome maturation [43], was significantly decreased in pSUPER-siPrxⅢ cells after H/R compared to pSUPER controls (Fig. 4A). Consistently, confocal imaging of GFP-LC3 revealed a marked reduction in puncta formation in PrxⅢ-depleted cells, whereas GFP-LC3 puncta markedly increased in pSUPER cells following H/R (Fig. 4B). These findings suggest impaired autophagosome formation in the absence of PrxⅢ.

To assess whether these defects extend to autophagic flux, we measured levels of SQSTM1/p62, a substrate degraded during autolysosome formation [44,45]. p62 protein expression decreased after H/R in pSUPER cells, consistent with efficient autophagic flux, whereas it accumulated in pSUPER-siPrxⅢ cells (Fig. 4A), indicating impaired autophagic progression.

To further visualize autophagic flux, cells were transduced with a tandem fluorescent-tagged mCherry-GFP-LC3 construct. In this system, yellow puncta represent autophagosomes (GFP and mCherry dual signal), while red-only puncta represent autolysosomes, as GFP is quenched in the acidic lysosomal environment [34]. Following H/R, the number of autolysosomes (red puncta) increased in control cells, while this increase was significantly blunted in PrxⅢ-deficient cells (Fig. 4C), reinforcing the conclusion that autophagic flux is impaired in the absence of PrxⅢ.

We next examined the expression of mitophagy-related proteins, including Parkin and BNIP3, which are critical regulators of mitochondrial clearance under stress conditions [46,47]. Both proteins were upregulated under basal conditions in PrxⅢ-deficient cells, suggesting a compensatory response to elevated mitochondrial stress. However, following H/R, the expression of both Parkin and BNIP3 increased in control cells but was reduced in pSUPER-siPrxⅢ cells (Fig. 4D), supporting the notion of defective mitophagy in the absence of PrxⅢ.

Collectively, these findings demonstrate that PrxⅢ depletion disrupts both autophagosome formation and downstream autophagic flux under H/R conditions in cardiomyocytes.

### 3.5 PrxⅢ depletion impairs lysosome function and autophagosome–lysosome fusion under H/R stress in cardiomyocytes

To further investigate the mechanism underlying impaired autophagic flux in PrxⅢ-deficient cells, we examined lysosomal function, as this is a key determinant of autolysosome formation and substrate clearance. Given the observed reduction in autolysosome formation in PrxⅢ-depleted cells (Fig. 4C), we hypothesized that lysosomal dysfunction might contribute to defective autophagy under H/R stress. Recent studies have shown that elevated ROS can compromise lysosomal activity and interfere with autophagosome–lysosome fusion [48,49].

**Fig. 5:**
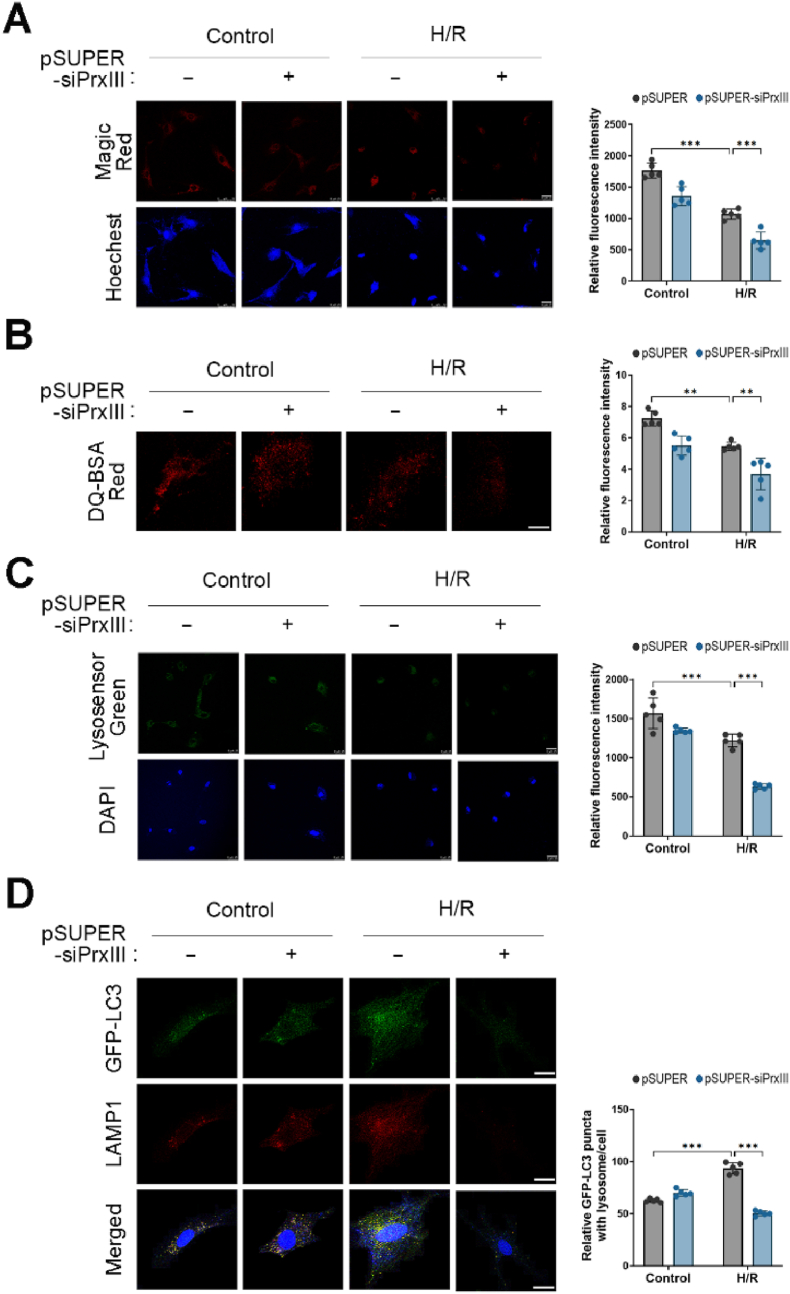
PrxⅢ depletion inhibits lysosome function and fusion with autophagosomes under H/R stress in cardiomyocytes. pSUPER and pSUPER-siPrxⅢ cells were subjected to 1 h of hypoxia followed by 4 h of reoxygenation (H/R). (A) Lysosomal protease activity was assessed using Magic Red (red), and nuclei were counterstained with Hoechst (blue). Scale bar, 25 μm. (B, C) Lysosomal acidity was measured by staining with (B) DQ-BSA Red (red; scale bar, 10 μm) and (C) LysoSensor Green DND-189 (green; scale bar, 25 μm). Nuclei were stained with DAPI (blue). (D) Cells were transduced with GFP-LC3 adenovirus for 24 h, followed by H/R treatment. After fixation, cells were immunostained for LAMP1 (red), and nuclei were counterstained with DAPI (blue). Colocalization of GFP-LC3 and LAMP1 was analyzed by confocal microscopy. Scale bar, 10 μm. (A-D) Five randomly selected microscopic fields per sample were analyzed and combined to represent one biological replicate. All data are presented as mean ± S.D. (n = 5). Statistical significance was determined using two-way ANOVA followed by Bonferroni's post hoc test. ∗∗p < 0.01 and ∗∗∗p < 0.001.

We first assessed lysosomal protease activity using Magic Red, a fluorogenic substrate for cathepsin B. Fluorescence intensity decreased after H/R stress and was further reduced in pSUPER-siPrxⅢ cells compared to controls, indicating attenuated lysosomal proteolytic capacity (Fig. 5A).

To evaluate lysosomal acidity, we employed two pH-sensitive fluorescent probes: DQ-BSA Red, which fluoresces upon proteolysis in acidic compartments, and LysoSensor Green, which accumulates in acidic lysosomes. Both signals were reduced after H/R and more prominently suppressed in PrxⅢ-depleted cells (Fig. 5B and C), indicating that H/R stress impairs lysosomal acidification, and that this defect is exacerbated by PrxⅢ depletion.

Finally, to assess autophagosome–lysosome fusion, we visualized the co-localization of GFP-LC3 (autophagosomes) and LAMP1 (lysosomes) by immunofluorescence microscopy. The number of yellow puncta, indicating successful fusion events, increased after H/R in control cells but remained low in PrxⅢ-deficient cells (Fig. 5D), suggesting that PrxⅢ is required for efficient autophagosome–lysosome fusion during oxidative stress.

These data collectively demonstrate that PrxⅢ deficiency compromises lysosomal function and fusion with autophagosomes, thereby contributing to autophagic flux impairment in cardiomyocytes subjected to H/R injury.

### 3.6 PrxⅢ depletion exacerbates apoptosis after H/R injury in cardiomyocytes

**Fig. 6:**
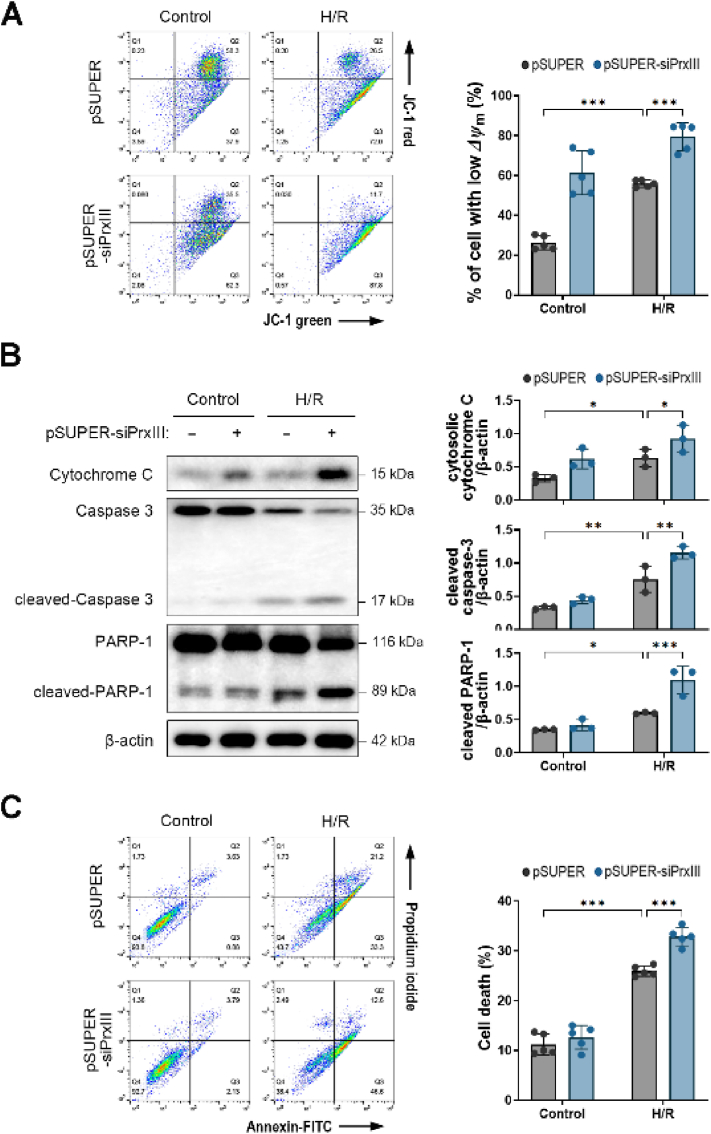
PrxⅢ depletion increases H/R stress-induced apoptotic cell death in cardiomyocytes. (A) Mitochondrial membrane potential (*ΔΨ*_m_) was measured by flow cytometry using JC-1 staining following 1 h of hypoxia and 4 h of reoxygenation (H/R). The percentage of cells with low *ΔΨ*_m_ (JC-1 green high/red low) was quantified. (B) Western blot analysis of cytosolic cytochrome C, cleaved caspase-3, and cleaved PARP-1 was performed in pSUPER and pSUPER-siPrxⅢ cells after H/R. β-actin was used as the loading control. Densitometric quantification of protein expression was performed from three independent biological replicates (n = 3). (C) Apoptotic cell death was assessed by Annexin V-FITC and propidium iodide (PI) double staining followed by flow cytometric analysis after 1 h of hypoxia and 8 h of reoxygenation. Quantification of Annexin V and/or PI-positive cells is shown. Flow cytometry-based data (A and C) represent five independent biological replicates (n = 5). All data are presented as mean ± S.D. Statistical significance was determined using two-way ANOVA followed by Bonferroni's post hoc test. ∗p < 0.05, ∗∗p < 0.01, and ∗∗∗p < 0.001.

To investigate whether PrxⅢ deficiency sensitizes cardiomyocytes to apoptotic cell death following H/R stress, we first assessed *ΔΨ*_m_ using JC-1 dye. A significant reduction in *ΔΨ*_m_ was observed after H/R treatment, and this depolarization was further enhanced in PrxⅢ-deficient cells compared to controls (Fig. 6A). We next examined the activation of mitochondria-dependent apoptotic signaling. Western blot analysis revealed increased levels of cytosolic cytochrome C, cleaved caspase-3, and cleaved PARP-1 in PrxⅢ-depleted cardiomyocytes following H/R stress, indicating enhanced activation of apoptotic signaling pathways (Fig. 6B).

To directly quantify apoptotic cell death, we performed annexin V-FITC and PI staining followed by flow cytometric analysis. To determine the optimal reoxygenation duration for this assay, we first conducted a time-course analysis, which revealed a progressive increase in apoptotic cell death with longer reoxygenation periods, plateauing at 8 h post-hypoxia (Supplementary Fig. S1B). Based on these findings, we selected 8 h of reoxygenation as the standard condition for subsequent apoptotic assessments. Under this condition, the percentage of annexin V- and/or PI-positive cells was significantly higher in PrxⅢ-deficient cardiomyocytes compared to controls (Fig. 6C). These results demonstrate that PrxⅢ protects cardiomyocytes against H/R-induced mitochondrial dysfunction and apoptosis by preserving *ΔΨ*_m_ and limiting the activation of intrinsic apoptotic signaling cascades.

### 3.7 Reconstitution of PrxⅢ or mitochondria-targeted catalase attenuates H/R-induced mitochondrial H_2_O_2_ accumulation in PrxⅢ-deficient cardiomyocytes

**Fig. 7:**
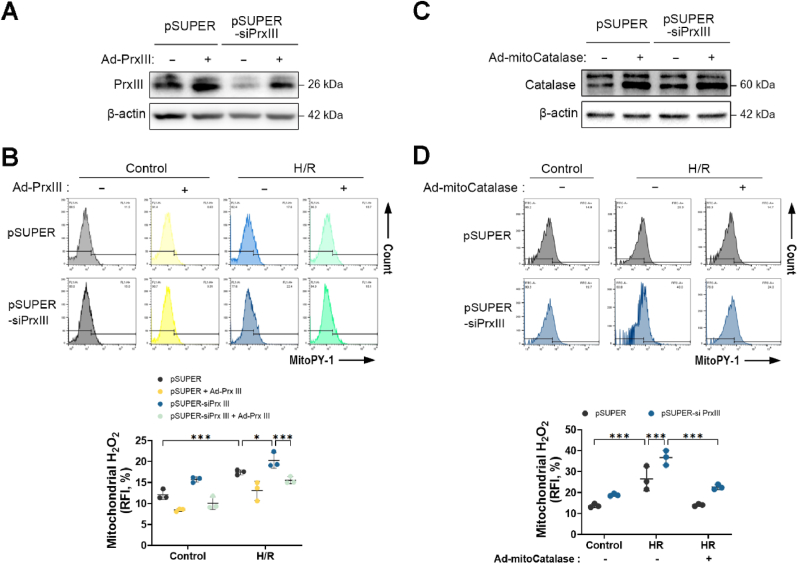
Reconstitution of PrxⅢ or mitochondrial catalase attenuates H/R-induced mitochondrial H_2_O_2_ accumulation in PrxⅢ-deficient cardiomyocytes. (A) pSUPER and pSUPER-siPrxⅢ cells were transduced with Ad-PrxⅢ or control Ad-Stuffer (MOI = 100) for 24 h, followed by 1 h of hypoxia and 4 h of reoxygenation (H/R). Reconstitution of PrxⅢ expression was confirmed by immunoblotting. (B) Mitochondrial H_2_O_2_ levels were measured using MitoPY-1 staining and flow cytometric analysis following Ad-PrxⅢ transduction and H/R treatment. (C) pSUPER and pSUPER-siPrxⅢ cells were transduced with Ad-mito-catalase (MOI = 100) for 24 h, and catalase expression was confirmed by immunoblotting. (D) Mitochondrial H_2_O_2_ levels were evaluated after Ad-mitoCatalase transduction and H/R using MitoPY-1 staining and flow cytometry. All data are presented as mean ± S.D. (n = 5). Statistical significance was determined using two-way ANOVA followed by Bonferroni's post hoc test. ∗p < 0.05 and ∗∗∗p < 0.001.

To further validate the role of PrxⅢ in regulating mitochondrial H_2_O_2_ levels under H/R stress, we employed adenoviral-mediated overexpression of human PrxⅢ in both pSUPER and pSUPER-siPrxⅢ H9c2 cells. Efficient overexpression of PrxⅢ was confirmed by immunoblotting (Fig. 7A). Reconstitution of PrxⅢ significantly reduced mitochondrial H_2_O_2_ accumulation after H/R exposure in PrxⅢ-deficient cells, as measured by MitoPY-1 fluorescence (Fig. 7B).

To confirm that the observed fluorescence reflected mitochondrial H_2_O_2_, we utilized adenoviral delivery of mitoCatalase, an established enzymatic scavenger of mitochondrial H_2_O_2_ [50]. Successful expression of mitoCatalase was validated by Western blot analysis (Fig. 7C). Overexpression of mitoCatalase substantially suppressed mitochondrial H_2_O_2_ levels following H/R in both PrxⅢ-sufficient and PrxⅢ-deficient cells (Fig. 7D).

These findings reinforce the role of PrxⅢ as a key regulator of mitochondrial redox homeostasis and validate the specificity of mitochondrial H_2_O_2_ measurements using MitoPY-1.

### 3.8 PrxⅢ reconstitution alleviates mitochondrial damage and apoptotic cell death induced by H/R in PrxⅢ-deficient cardiomyocytes

**Fig. 8:**
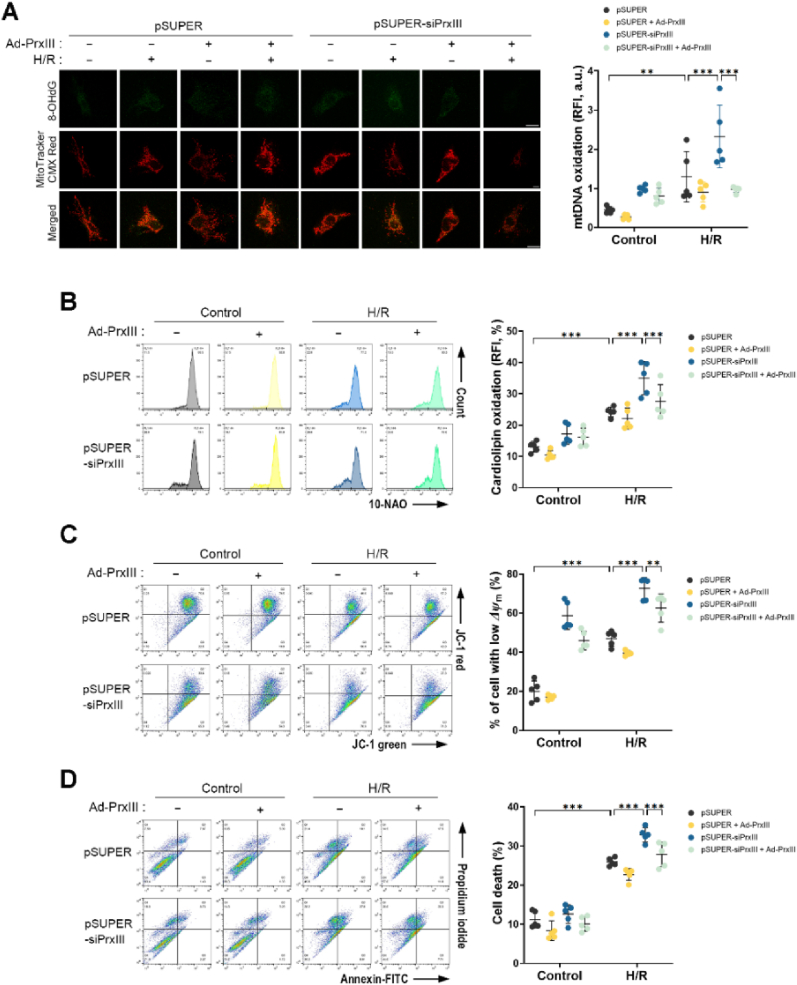
Reconstitution of PrxⅢ mitigates oxidative stress and apoptotic cell death induced by H/R in PrxⅢ-deficient cardiomyocytes. pSUPER and pSUPER-siPrxⅢ cells were transduced with Ad-PrxⅢ or control Ad-Stuffer (MOI = 100) for 24 h, followed by 1 h of hypoxia and 4 h of reoxygenation (H/R). (A) Mitochondrial DNA (mtDNA) oxidation was visualized by immunofluorescence staining for 8-OHdG (green), with mitochondria labeled using MitoTracker CMX Red (red). Co-localization (yellow) indicates oxidized mtDNA. Images were captured by confocal microscopy and analyzed using Fiji software. Five randomly selected microscopic fields per sample were analyzed and combined to represent one biological replicate. Scale bars, 10 μm. (B) Mitochondrial lipid peroxidation was measured by flow cytometry using 10-N-nonylacridine orange (10-NAO) as a cardiolipin oxidation probe. (C) Mitochondrial membrane potential (ΔΨm) was assessed by JC-1 staining and quantified by flow cytometry as the percentage of cells with low ΔΨm. (D) Apoptotic cell death was analyzed by Annexin V-FITC and propidium iodide staining after 1 h of hypoxia followed by 8 h of reoxygenation. Flow cytometry-based assays (B–D) were conducted with five independent biological replicates (n = 5). All data are presented as mean ± S.D. Statistical significance was determined using two-way ANOVA followed by Bonferroni's post hoc test. ∗∗p < 0.01 and ∗∗∗p < 0.001.

To determine whether restoration of PrxⅢ expression could mitigate mitochondrial damage under oxidative stress, we reconstituted PrxⅢ in pSUPER-siPrxⅢ cardiomyocytes via adenoviral transduction. Ectopic PrxⅢ expression significantly attenuated H/R-induced mitochondrial macromolecular oxidative damage, as evidenced by reduced mtDNA oxidation (8-OHdG staining) and decreased cardiolipin peroxidation (10-NAO fluorescence) (Fig. 8A and B). In addition, PrxⅢ reconstitution restored *ΔΨ*_m_, which was markedly depolarized following H/R treatment in PrxⅢ-depleted cells, as assessed by JC-1 staining (Fig. 8C). Consistent with these mitochondrial protective effects, Annexin V-FITC/PI staining revealed reduced apoptotic cell death in PrxⅢ-restored cells following H/R stress (Fig. 8D). These results indicate that PrxⅢ plays a critical role in protecting cardiomyocytes from H/R-induced mitochondrial oxidative damage and apoptosis.

### 3.9 PrxⅢ reconstitution restores mitochondrial morphology, autophagic flux, and lysosome function impaired by H/R injury in PrxⅢ-deficient cardiomyocytes

**Fig. 9:**
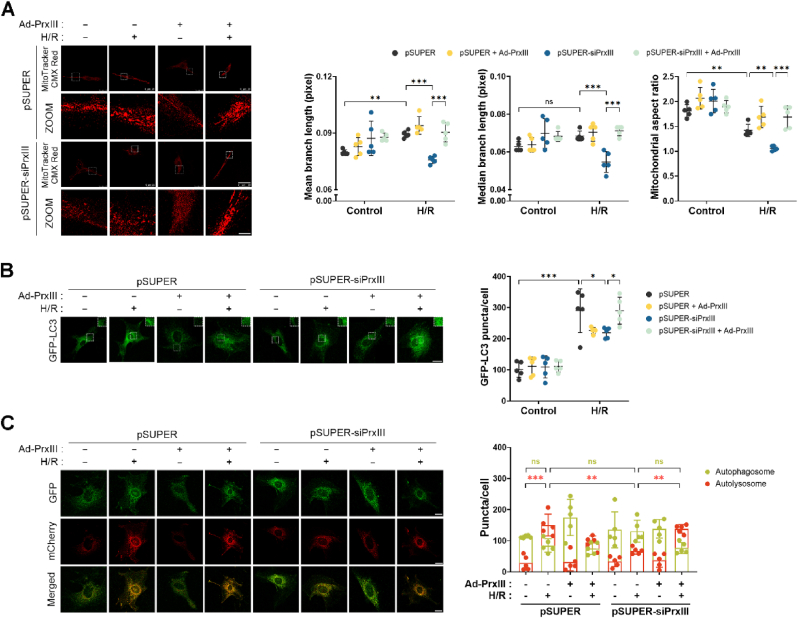
Reconstitution of PrxⅢ restores mitochondrial dynamics and autophagosome formation impaired by H/R stress in PrxⅢ-deficient cardiomyocytes. (A) Mitochondrial morphology was assessed in pSUPER and pSUPER-siPrxⅢ cells transduced with Ad-PrxⅢ or Ad-Stuffer (MOI = 100) for 24 h, followed by 1 h hypoxia and 4 h reoxygenation (H/R). Cells were stained with MitoTracker CMX Red and imaged by confocal microscopy. Zoomed panels highlight boxed regions. Mitochondrial morphology was analyzed using Fiji software with the MiNA plugin. Scale bars, 50 μm (main) and 10 μm (zoom). (B) Cells were transduced with Ad-PrxⅢ and GFP-LC3 adenovirus for 24 h and subjected to H/R. GFP-LC3 puncta were visualized by confocal microscopy and quantified using ImageJ. Scale bars, 10 μm (main) and 2 μm (zoom). (C) Cells transduced with Ad-PrxIII and mCherry-GFP-LC3 adenovirus were subjected to H/R, and confocal microscopy was used to assess autophagosomes (yellow puncta) and autolysosomes (red puncta). Scale bars, 25 μm. (A-C) Five randomly selected microscopic fields per sample were analyzed and combined to represent one biological replicate. All data are presented as mean ± S.D. (n = 5). Statistical significance was determined using two-way ANOVA followed by Bonferroni's post hoc test. ∗p < 0.05, ∗∗p < 0.01, ∗∗∗p < 0.001, and ns, p > 0.05 (not significant).

We next investigated whether PrxⅢ reconstitution could reverse H/R-induced impairments in mitochondrial dynamics and the autophagy–lysosome pathway. Confocal microscopy using MitoTracker Red staining revealed improved mitochondrial morphology with increased branch length in PrxⅢ-reconstituted cells, compared to the fragmented phenotype seen in PrxⅢ-depleted cells following H/R (Fig. 9A). This was further supported by aspect ratio analysis, which showed a shift toward more elongated mitochondria (Fig. 9A, far right).

To assess autophagic activity, we first examined GFP-LC3 puncta formation, which was significantly increased after PrxⅢ overexpression, indicating enhanced autophagosome formation (Fig. 9B). In addition, mCherry-GFP-LC3 tandem fluorescence analysis revealed restored autophagic flux, as shown by increased red puncta corresponding to autolysosomes (Fig. 9C).

**Fig. 10:**
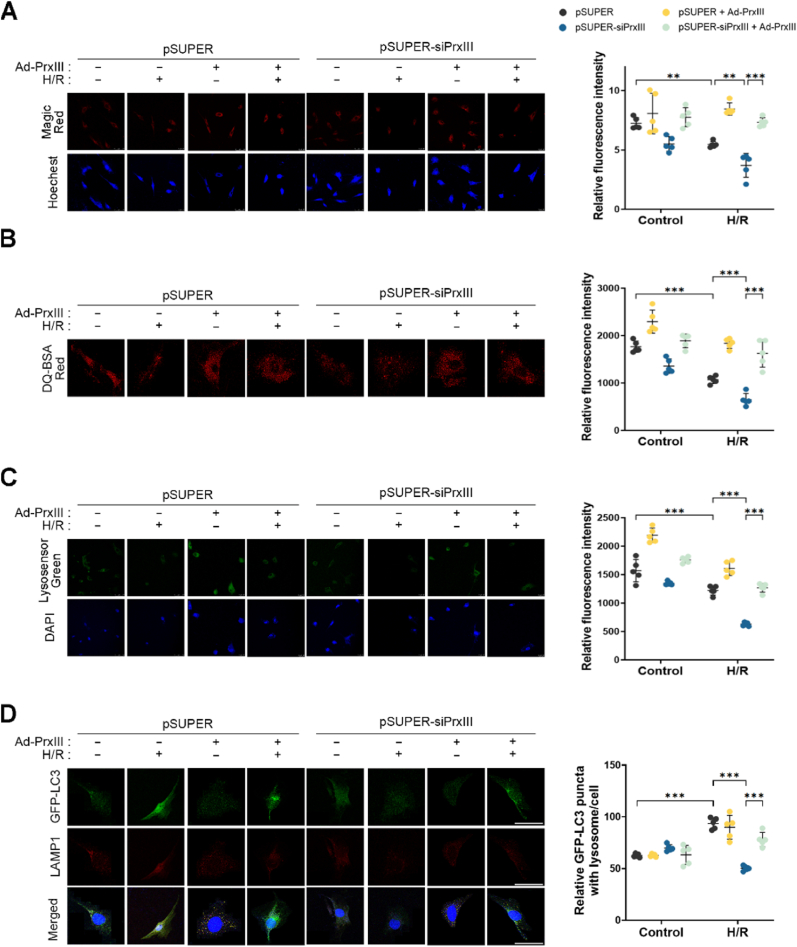
Reconstitution of PrxⅢ restores lysosomal function and autophagosome–lysosome fusion impaired by H/R in PrxⅢ-deficient cardiomyocytes. pSUPER and pSUPER-siPrxⅢ cells were transduced with Ad-PrxⅢ or Ad-Stuffer (MOI = 100) for 24 h, followed by 1 h hypoxia and 4 h reoxygenation (H/R). (A) Cathepsin B activity was measured using the Magic Red fluorescent probe (red), and nuclei were counterstained with Hoechst (blue). (B) Lysosomal proteolytic activity was assessed using the self-quenched fluorescent substrate DQ-BSA Red. (C) Lysosomal acidity was evaluated using LysoSensor Green DND-189 (green), with DAPI (blue) used for nuclear staining. (D) For analysis of autophagosome–lysosome fusion, cells were co-transduced with GFP-LC3 and Ad-PrxⅢ, fixed after H/R stress, and immunostained for LAMP1 (red). Colocalization between autophagosomes and lysosomes was visualized as yellow puncta in merged images. All images were acquired by confocal microscopy. Scale bars: 25 μm (Magic Red, LysoSensor Green, and GFP-LC3/LAMP1), 10 μm (DQ-BSA). Fluorescence intensities and colocalized puncta were quantified using Fiji software. Five randomly selected microscopic fields per sample were analyzed and combined to represent one biological replicate (n = 5). All data are presented as mean ± S.D. Statistical significance was determined using two-way ANOVA followed by Bonferroni's post hoc test. ∗∗p < 0.01 and ∗∗∗p < 0.001.

Complementary analyses of lysosomal function demonstrated that PrxⅢ reconstitution restored both proteolytic activities, as indicated by Magic Red fluorescence (Fig. 10A), and lysosomal acidity, as evidenced by increased fluorescence of pH-sensitive dyes (Fig. 10B and C). Moreover, co-localization of GFP-LC3 with LAMP1, a marker of autophagosome–lysosome fusion, was also significantly increased in PrxⅢ-reconstituted cells (Fig. 10D), further supporting recovery of autophagic clearance.

Together, these findings demonstrate that PrxⅢ is essential for maintaining MQC, sustaining autophagic flux, and preserving lysosomal function during H/R stress.

### 3.10 PrxⅢ deficiency aggravates mitochondrial oxidative stress and mitophagy impairment in primary cardiomyocytes and ischemic mouse hearts

**Fig. 11:**
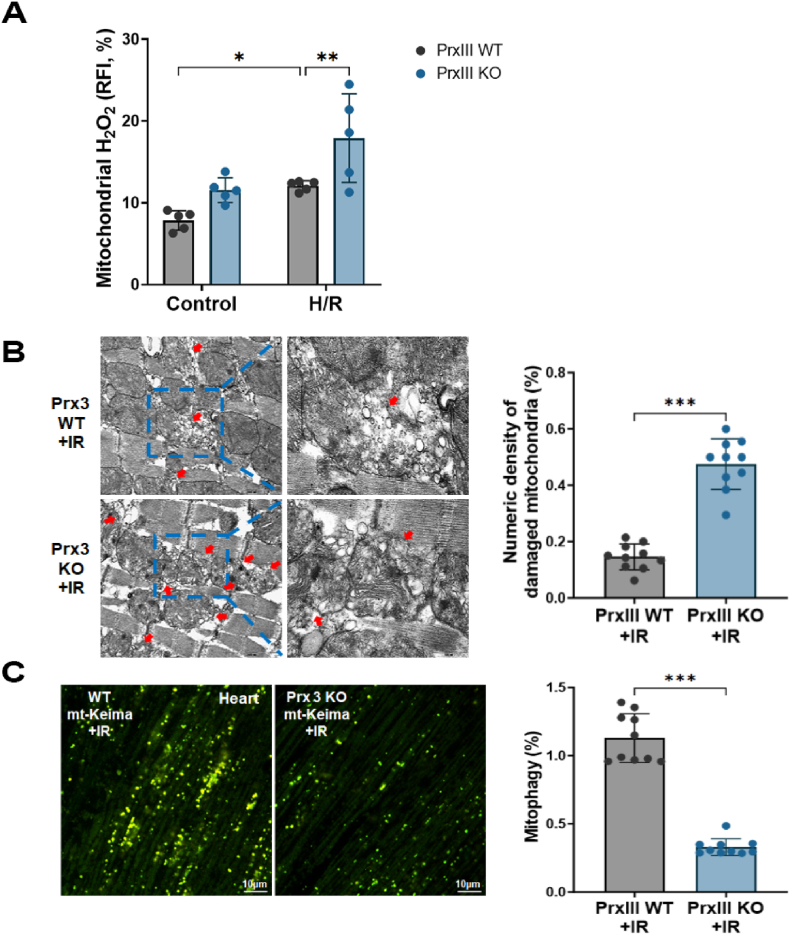
PrxⅢ deficiency exacerbates mitochondrial ROS production, structural damage, and mitophagy impairment in cardiomyocytes and ischemic hearts. (A) Mitochondrial H_2_O_2_ levels were measured in primary cardiomyocytes isolated from wild-type (PrxⅢ WT) and PrxⅢ knockout (PrxⅢ KO) neonatal mice subjected to hypoxia (1 h) and reoxygenation (4 h). MitoPY-1 staining was used to detect mitochondrial H_2_O_2_, and fluorescence was quantified by flow cytometry. Data are expressed as mean ± S.D. from independent experiments (n = 5). (B) Transmission electron microscopy (TEM) images show mitochondrial ultrastructure in heart tissue collected 1 day after myocardial infarction (MI) from 10-week-old PrxⅢ WT and PrxⅢ KO mice. Blue dashed boxes indicate zoomed regions; red arrows highlight structurally damaged mitochondria. Scale bars, 2 μm (overview) and 0.5 μm (zoom). Quantification of damaged mitochondria was performed from five mice per group (n = 5). (C) Mitophagy was assessed in heart tissues from PrxⅢ WT and PrxⅢ KO mt-Keima mice 1 day after MI. Representative confocal microscopy images are shown. The percentage of red-to-green shifted mt-Keima fluorescence was quantified to measure mitophagy. Scale bar, 10 μm. Data are expressed as mean ± S.D. from five biologically independent mice (n = 5). All data are presented as mean ± S.D. Statistical significance was determined using two-way ANOVA followed by Bonferroni's post hoc test. ∗p < 0.05, ∗∗p < 0.01, and ∗∗∗p < 0.001.

To extend the relevance of our findings beyond the H9c2 cell model, we assessed the impact of PrxⅢ deficiency in primary cardiomyocytes and *in vivo* heart tissues. Primary cardiomyocytes isolated from neonatal PrxⅢ WT and KO mice were subjected to H/R stress. Consistent with our earlier *in vitro* results, mitochondrial H_2_O_2_ levels were significantly elevated under H/R conditions, with a markedly greater increase observed in PrxⅢ KO cells compared to WT controls (Fig. 11A). These findings confirm that PrxⅢ loss enhances mitochondrial oxidative stress in primary cardiomyocytes.

To further explore the physiological relevance in an *in vivo* setting, we employed a murine model of myocardial I/R injury using 10-week-old PrxⅢ WT and KO mice. Electron microscopy of heart tissue collected 24 h after I/R revealed a significantly higher density of structurally damaged mitochondria in PrxⅢ KO hearts, consistent with increased mitochondrial vulnerability (Fig. 11B).

We next evaluated mitophagy using mt-Keima reporter mice. PrxⅢ KO hearts exhibited a marked reduction in red-to-green fluorescence shift following I/R, indicating impaired mitophagy (Fig. 11C). These *in vivo* findings are in line with our previous cellular observations of disrupted autophagic flux and downregulation of mitophagy regulators in PrxⅢ-deficient cells (see Fig. 4). Together, these results reinforce the role of PrxⅢ as a key mitochondrial antioxidant that safeguards mitophagy competence and mitochondrial integrity under ischemic stress.

## 4 Discussion

Cardiac I/R injury is a multifactorial pathology characterized by oxidative stress and mitochondrial dysfunction. Among the ROS generated during reperfusion, H_2_O_2_ plays a central pathogenic role due to its membrane-permeable and signaling-capable nature [51]. In this study, we investigated the cardioprotective role of mitochondrial PrxⅢ against H/R-induced oxidative damage and explored its functional relationship with MQC.

Our findings expand upon previous reports, such as that by Nonn et al. [27], which demonstrated the anti-apoptotic role of PrxⅢ in cancer cells. Here, we extend its protective function into the cardiac setting, confirming that PrxⅢ effectively reduces mitochondrial H_2_O_2_ levels, preserves organelle integrity, and suppresses apoptosis in cardiomyocytes under oxidative stress.

While ROS play both signaling and damaging roles, excessive mitochondrial H_2_O_2_ disrupts MQC mechanisms including mitochondrial dynamics, mitophagy, and lysosome function. Notably, we observed that electrically neutral H_2_O_2_ diffused from mitochondria to the cytosol, thereby amplifying oxidative damage beyond the mitochondrial compartment (Fig. 1). This suggests that PrxⅢ not only limits mitochondrial oxidative stress but also serves as a barrier to secondary cytosolic ROS propagation.

Mitochondria dynamically regulate their morphology through fusion and fission processes, which are essential components of MQC. Interestingly, under normoxic conditions, PrxⅢ-deficient cardiomyocytes exhibited increased expression of fusion proteins Mfn1 and OPA1, suggesting a compensatory response to mild oxidative stress. This adaptive mechanism may reflect a lower threshold for redox-induced mitochondrial fusion aimed at preserving organelle function. However, under severe oxidative stress such as H/R, these fusion-related proteins were significantly downregulated, resulting in pronounced mitochondrial fragmentation (Fig. 3). This biphasic response highlights the presence of distinct adaptive thresholds governing mitochondrial dynamics. Under moderate oxidative stress, fusion is promoted to counteract damage, whereas excessive oxidative burden overwhelms fusion capacity and shifts the balance toward fission. These findings are in agreement with previous studies reporting context-dependent regulation of mitochondrial dynamics in response to redox cues [22,24,52].

Mitochondrial fission is largely orchestrated by Drp1. Our results revealed that phosphorylation at the inhibitory site Ser637 was markedly reduced in PrxⅢ-deficient cells following H/R, facilitating Drp1 translocation to mitochondria and promoting fission. In contrast, phosphorylation at Ser616—typically associated with Drp1 activation—remained unchanged (Fig. 3C). These data suggest that mitochondrial fragmentation observed in our model primarily stems from reduced inhibitory control rather than enhanced activation of Drp1, further supporting the idea that oxidative stress drives mitochondrial fission via derepression mechanisms. This mechanism aligns with prior findings implicating Ser637 dephosphorylation as a key redox-sensitive switch in mitochondrial dynamics [53].

Mitophagy, a selective form of autophagy, is essential for MQC as it ensures the removal of dysfunctional mitochondria. It proceeds through three key steps—initiation of phagophore formation, recognition of damaged mitochondria, and fusion with lysosomes—all of which are sensitive to redox imbalance. ROS have been shown to impair multiple stages of mitophagy. For example, oxidation of autophagy-related proteins by ROS interferes with phagophore elongation and LC3 lipidation, thereby disrupting the early stages of autophagosome formation [54].

Damaged mitochondria are typically recognized and sequestered via adaptor- and receptor-mediated mechanisms. The PINK1/Parkin pathway represents a key adaptor-based mechanism. Consistent with our previous findings demonstrating that PrxⅢ interacts with and stabilizes PINK1 under stress conditions [35], the present study confirmed that Parkin expression was elevated under moderate oxidative stress (i.e., PrxⅢ knockdown or H/R in wild-type cells), suggesting initiation of mitophagic clearance. However, under conditions of combined PrxⅢ deficiency and H/R stress—when mitochondrial H_2_O_2_ reached critical levels—Parkin expression markedly declined. This supports the idea that excessive ROS suppress mitophagy signaling, as previously proposed [55,56].

BNIP3, a representative receptor in the LC3-interacting region (LIR)-mediated pathway, showed a similar biphasic pattern. While BNIP3 increased after H/R in control cells, its expression was reduced in PrxⅢ-deficient cardiomyocytes, again implying that excessive mitochondrial ROS blocks mitophagy at the recognition stage. These results suggest that mitochondrial H_2_O_2_ levels act as a rheostat: low to moderate ROS levels induce mitophagy as a compensatory survival mechanism, whereas excessive ROS inhibit this clearance process. This is consistent with previous studies showing ROS-sensitive biphasic regulation of mitophagy [57].

To verify these findings beyond protein expression levels, we employed mt-Keima reporter mice to directly monitor mitophagy *in vivo*. The pH-sensitive fluorescence shift of mt-Keima from green (neutral pH) to red (acidic lysosomal pH) enables reliable tracking of mitochondrial degradation. We observed significantly attenuated red-to-green conversion in PrxⅢ knockout mouse hearts following I/R injury (Fig. 11C), indicating that PrxⅢ is indispensable for maintaining mitophagy flux under physiological ischemic stress conditions.

Finally, we examined whether the third step of mitophagy—fusion of mitophagosomes with lysosomes to form mitolysosomes—was also impaired. Lysosomal acidity and cathepsin activity were both significantly compromised in PrxⅢ-deficient cardiomyocytes under H/R, impairing autophagic clearance. Co-localization of LC3-positive autophagosomes with LAMP1-positive lysosomes was also reduced. Restoration of PrxⅢ rescued these defects, demonstrating that PrxⅢ not only regulates mitochondrial redox balance but also preserves lysosome function necessary for completion of mitophagy.

Importantly, our *in vivo* and primary cardiomyocyte data validated these mechanisms under physiological conditions. Primary cardiomyocytes isolated from PrxⅢ KO mice exhibited increased mitochondrial ROS accumulation following H/R stress, and adult KO mice subjected to myocardial I/R injury showed elevated mitochondrial fragmentation and defective mitophagy (Fig. 11A–C). The concordant findings from both H9c2 cells and PrxⅢ KO mice provide strong evidence that PrxⅢ is a critical regulator of redox homeostasis and MQC during cardiac stress.

In conclusion, our study demonstrates that PrxⅢ is a critical regulator of mitochondrial redox homeostasis and MQC in cardiomyocytes under H/R stress. By limiting mitochondrial H_2_O_2_ accumulation, PrxⅢ preserves mitochondrial morphology, supports mitophagic clearance, and maintains lysosomal integrity. These findings provide mechanistic insight into how mitochondrial antioxidants shape the cardiomyocyte response to ischemic injury and suggest that enhancing PrxⅢ function may represent a novel therapeutic approach in I/R-related cardiac disease.

## 5 Conclusions

This study demonstrates that PrxⅢ is a critical determinant of mitochondrial redox homeostasis and cardiomyocyte survival under H/R stress. By effectively scavenging mitochondrial H_2_O_2_, PrxⅢ preserves mitochondrial structure, supports MQC, and maintains lysosomal function. PrxⅢ deficiency leads to excessive mitochondrial and cytosolic oxidative stress, resulting in disrupted mitochondrial dynamics, impaired mitophagic flux, and lysosomal dysfunction. These effects collectively contribute to mitochondrial damage and apoptosis. Restoration of PrxⅢ expression reverses these defects, highlighting its essential role in protecting cardiomyocytes from H/R-induced injury. Our findings suggest that therapeutic targeting of PrxⅢ may offer a promising strategy to combat ischemia/reperfusion-related cardiac damage by reinforcing the antioxidant and organelle-protective machinery of mitochondria.

## CRediT authorship contribution statement

**Ji Won Park:** Conceptualization, Data curation, Formal analysis, Methodology, Software, Visualization, Writing – original draft, Writing – review & editing. **Seong Keun Sonn:** Conceptualization, Formal analysis, Investigation, Methodology, Software. **Byung-Hoon Lee:** Methodology, Validation. **Goo Taeg Oh:** Methodology, Resources, Validation. **Tong-Shin Chang:** Conceptualization, Funding acquisition, Methodology, Project administration, Resources, Supervision.

## Funding

This work was supported by the 10.13039/501100003725National Research Foundation of Korea (NRF) grant funded by the 10.13039/501100014188Ministry of Science and ICT (10.13039/501100014188MSIT) (NRF-2020R1A2C1006443), the Basic Science Research Program through the 10.13039/501100003725National Research Foundation of Korea funded by the 10.13039/100009950Ministry of Education (NRF-2022R1A6A1A03046247), and the Brain Korea 21 Plus Program funded by the Korean government (10.13039/100009122MOE) in the Republic of Korea.

## Declaration of competing interest

The authors declare that they have no known competing financial interests or personal relationships that could have appeared to influence the work reported in this paper.

## Appendix A Supplementary data

Supplementary Figure S1. Optimization of reoxygenation duration following hypoxic stress in H9c2 cardiomyocytes.H9c2 cells were subjected to 1 h of hypoxia followed by reoxygenation for the indicated durations. (A) Mitochondrial H_2_O_2_ was measured using MitoPY-1 staining and flow cytometry. (B) Cell death was assessed by Annexin V-FITC and propidium iodide (PI) co-staining, followed by flow cytometric analysis. All data are presented as mean ± S.D. from independent experiments (n = 3). ∗∗p < 0.01 and ∗∗∗p < 0.001 versus control (0 h) by one-way ANOVA followed by Dunnett's post hoc test.
