# Supplementary material for: Peroxiredoxin Ⅲ mitigates mitochondrial H2O2-mediated damage and supports quality control in cardiomyocytes under hypoxia-reoxygenation stress
Source: Redox Biol. 2025 Aug 5;86:103799. doi: 10.1016/j.redox.2025.103799 (PMC12355573; doi:10.1016/j.redox.2025.103799)
Supplement: Multimedia component 1 [file mmc1.docx]

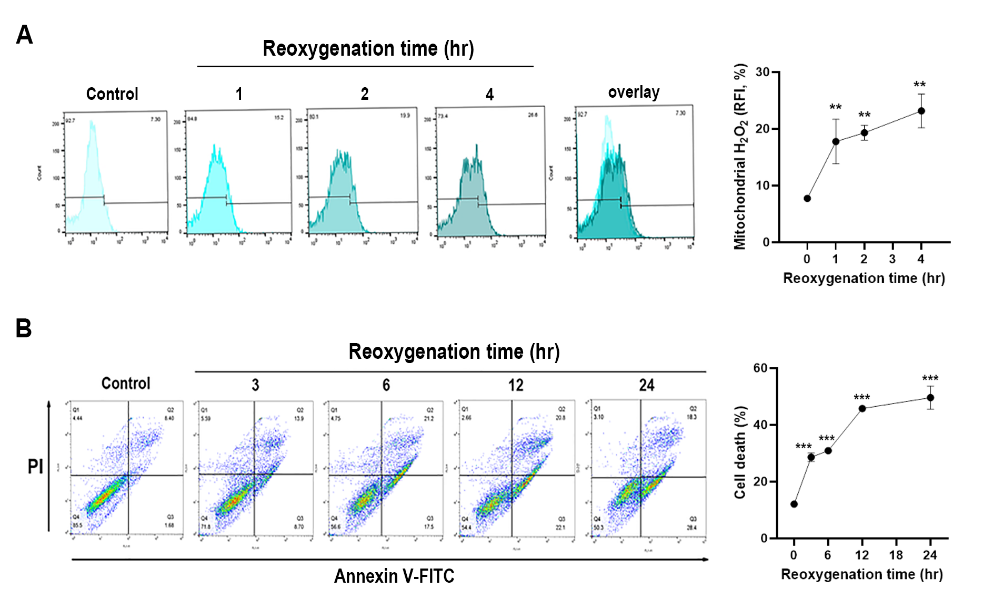


### Supplementary Figure S1. Optimization of reoxygenation duration following hypoxic stress in H9c2 cardiomyocytes. H9c2 cells were subjected to 1 h of hypoxia followed by reoxygenation for the indicated durations. (A) Mitochondrial H₂O₂ was measured using MitoPY-1 staining and flow cytometry. (B) Cell death was assessed by Annexin V-FITC and propidium iodide (PI) co-staining, followed by flow cytometric analysis. All data are presented as mean ± S.D. from independent experiments (n = 3). **p < 0.01 and ***p < 0.001 versus control (0 h) by one-way ANOVA followed by Dunnett’s post hoc test.
